# Larval Tolerance to Salinity in Three Species of Australian Anuran: An Indication of Saline Specialisation in *Litoria aurea*

**DOI:** 10.1371/journal.pone.0043427

**Published:** 2012-08-20

**Authors:** Brian D. Kearney, Phillip G. Byrne, Richard D. Reina

**Affiliations:** 1 Australian Centre for Biodiversity, School of Biological Sciences, Monash University, Clayton, Victoria, Australia; 2 School of Biological Sciences, Monash University, Clayton, Victoria, Australia; 3 The Institute for Conservation Biology and Environmental Management, University of Wollongong, Wollongong, New South Wales, Australia; University of Sao Paulo, Brazil

## Abstract

Recent anthropogenic influences on freshwater habitats are forcing anuran populations to rapidly adapt to high magnitude changes in environmental conditions or face local extinction. We examined the effects of ecologically relevant elevated salinity levels on larval growth, metamorphosis and survival of three species of Australian anuran; the spotted marsh frog (*Limnodynastes tasmaniensis*), the painted burrowing frog (*Neobatrachus sudelli*) and the green and golden bell frog *(Litoria aurea*), in order to better understand the responses of these animals to environmental change. Elevated salinity (16% seawater) negatively impacted on the survival of *L. tasmaniensis* (35% survival) and *N sudelli* (0% survival), while reduced salinity had a negative impact on *L. aurea*. (16% seawater: 85% survival; 0.4% seawater: 35% survival). *L. aurea* tadpoles survived in salinities much higher than previously reported for this species, indicating the potential for inter-populations differences in salinity tolerance. In *L. tasmaniensis* and *L. aurea,* development to metamorphosis was fastest in low and high salinity treatments suggesting it is advantageous for tadpoles to invest energy in development in both highly favourable and developmentally challenging environments. We propose that this response might either maximise potential lifetime fecundity when tadpoles experience favourable environments, or, facilitate a more rapid escape from pond environments where there is a reduced probability of survival.

## Introduction

Organisms are often forced to exist in habitats that undergo rapid and severe environmental change. Many organisms respond to these changes by evolving plastic responses in morphology, physiology and behaviour [1]. Reactions that allow the organism to grow and reproduce under variable environmental conditions will be favoured by natural selection, provided that the reactions are heritable and that the costs do not outweigh the benefits of the response [2]. However, if changes in environmental condition are of a greater magnitude than a species is able to tolerate, no adaptation can occur, resulting in an increased probability of local extinction, and an associated decline in species diversity [3].

Amphibians act as crucial herbivore, carnivore and prey species at varying points throughout their developmental cycle in both terrestrial and aquatic environments [4]. Amphibians are integral components of many ecosystems and their loss will have important impacts on various trophic levels, and ultimately reduce ecosystem stability and resilience [4]. Amphibian populations are in decline, and in Australia alone over 15 percent of frog species (33/217) are currently listed as extinct, endangered or vulnerable [5]. Proposed reasons for these declines have included increased exposure to ultra-violet radiation, acidification, climate change, impacts of introduced species, pathogens such as the virulent chytrid fungus disease, and changes to air and water quality [see review in 3]. In Australia, secondary salinisation is considered one of the greatest causes of degradation in freshwater aquatic systems and has been identified as a substantial threat to many freshwater taxa [6]. However, it is only in the last decade that the effects of salinity stress on Australian amphibians have received empirical attention [6], [7], [8], [9]. As a result, many of the physiological and ecological effects of increasing salinity on native Anuran populations remain unknown. Since European settlement of Australia, human induced secondary salinisation has become a pervasive environmental problem [10]. The main causes of secondary salinisation in the region have been the removal of natural deep-rooted vegetation in order to replace it with shallow-rooted agricultural plants, and an increase in irrigation practices that produce a discharge of saline wastewater [11]. These practices have resulted in rising ground-waters bringing salts to the surface, which are then deposited into rivers lakes and wetlands, either directly or through run-off water. The result has been an increase in salinity of many previously freshwater bodies (<0.2% seawater) to brackish levels (12% seawater) and above [6]. These changes could have substantial direct impacts on Australian amphibians.

Adult anurans possess several characteristics that make them particularly sensitive to increases in environmental salinity, including highly permeable skin, a dependence on both terrestrial and aquatic environments throughout their life cycle, small home ranges and a generally poor ability to osmoregulate [12]. Tadpoles and eggs are more susceptible than adults as they are restricted to the aquatic environment in which they develop and their osmoregulatory organs are still under development in the early larval stages, making them more likely to fail in unfavourable environments [13]. This susceptibility to changes in water quality is often compounded in species that oviposit in ephemeral water bodies because concentration of salts in these bodies will increase following evaporation [14]. Consequently, most amphibians actively avoid inhabiting and breeding in saline and brackish environments [15], although there are a few notable exceptions. Specifically, the crab-eating frog (*Fejervarya cancrivora*, formerly *Rana cancrivora*) of south-east Asia and the green toad (*Bufo viridis*) of Europe and the Middle East can tolerate salinities up to approximately 75% seawater [10]. In Australia, it remains unknown whether there are any salt-adapted anurans, but current salinities levels in wetlands are suspected to exceed the absolute tolerances of many species [16].

In order to better understand the ability of Australian frogs to tolerate elevated salinity we examined the effect of ecologically relevant salinity levels on larval growth, metamorphic timing and survival of three species of Australian anuran; the spotted marsh frog (*Limnodynastes tasmaniensis*), the painted burrowing frog (*Neobatrachus sudelli*) and the green and golden bell frog *(Litoria aurea*). *L. tasmaniensis* and *N. sudelli* are opportunistic breeders that will readily colonise temporary or permanent water bodies, including areas associated with human habitation that have experienced elevated salinity levels via secondary salinisation [6], [17]. *L. aurea* is a native hylid frog that is known to inhabit large coastal swamps that are often inundated with sea spray, and their tadpoles may therefore have a high degree of tolerance to elevated salinity [17]. By examining the embryonic and larval salinity tolerances in these species and investigating the developmental costs of a high tolerance to salinity, we will be able to better understand the how these animals are responding to environmental change.

## Materials and Methods

This study conformed to current Australian law and was conducted under Monash University animal ethics approval BSCI2009/14.

### Experimental Design

To examine the effects of elevated salinity on larval growth and survival, tadpoles of *L. aurea*, *N. sudelli* and *L. tasmaniensis* were reared under four salinity treatments: 0.4% seawater (sw), 4% sw, 10% sw and 16% sw (0.14 ppt, 1.4 ppt, 3.5 ppt and 5.6 ppt sea salt respectively). These salinities were chosen as they reflect the range of salinities that tadpoles experience in the wild [6], and are comparable with other studies of Australian anuran salinity tolerances [7]. Approximately four to seven days after hatching, eighty tadpoles of each species (between 4 to 7 clutches) were randomly allocated to the four treatments (i.e. 20 tadpoles per treatment) and monitored until either death or metamorphosis occurred. Tadpoles were housed individually to prevent confounding developmental impacts associated with intra-specific competition [18]. We examined the effects of the treatments on survival to metamorphosis, time to metamorphosis, and body size at metamorphosis (snout-vent length and mass). These are commonly used measures of anuran fitness [cf. 19].

### Husbandry Techniques

*Limnodynastes tasmaniensis* and *N. sudelli* egg clutches (approximately 1–2 days old) tadpoles (approximately 6–7 days old) were obtained from several fresh water ponds (less than 0.4% sw) in Beaufort and (37°26′S 143°23′E) Australia. *Litoria aurea* tadpoles were obtained from a captive freshwater colony originally obtained from a mosaic wetland in Eastern Victoria, where salinities ranged from fresh to highly brackish (0.4% sw to 15% sw). For all experimental treatments, laboratory grade filtered water (Millipore) was mixed with Ocean Nature Synthetic Sea Salt (Aquasonic; Wauchope, Australia) to achieve the desired salinity level. Sea salt was used to make salt solutions as sodium chloride does not provide the necessary ions for proper osmoregulation [20], and because it more closely reflects natural salts found in inland Victoria [21]. To reduce osmotic shock in the 4% sw, 10% sw, and 16% sw treatments, tadpoles were first placed in a half strength salt solution (2% sw, 5% sw and 8% sw respectively), which was then raised to full strength approximately 24 hours later. Animals were housed collectively for the first two days of salt treatment and then transferred into individual one litre (13 cm×13 cm×6 cm) containers holding 600 ml of treatment water. Treatment containers were then arranged randomly on benches in a laboratory. Water temperatures were maintained at 20°C (±2°C) and water levels and salinity was monitored twice weekly and adjusted as necessary to maintain consistent salt concentrations. Tadpoles were fed *ad libitum* on a mixture of frozen endive and *Spirulina* algal flakes (Nutrafin Max; West Yorkshire UK) and partial (≈70%) water changes were made once per week in order to prevent water fouling.

### Effect of Salinity on Tadpole Survival, Growth and Development

Survival of each tadpole was recorded daily until metamorphosis [Gosner stage 42-fore-limb emergence; 22]. The effect of salinity on tadpole growth was determined by measuring tadpole body length (snout to tail tip) once a week, using standardised overhead digital photographs. Due to the possible bias of large tadpoles being removed from the group as they completed metamorphosis, only measurements taken over the first ten weeks of development were analysed. To measure body length, tadpoles were transferred to a shallow plastic dish containing enough treatment water to completely cover the subject. A photograph was then taken using a digital camera attached to a Leica stereo microscope and body length measured (±0.1 mm) using Image J Image Processing Software (open source, version 1.42q). Measurements were calibrated against a standard scale present in each photograph. Within twelve hours of metamorphosis, each metamorph was removed from the treatment container, photographed, then blotted dry using paper towel and weighed to the nearest 0.01 g (Shimadzu balance, Kyoto, Japan).

### Statistical Analysis

To test the effect of the different salinity treatments on tadpole viability we compared offspring survival to metamorphosis, time (days) to metamorphosis and body size (snout-vent length and mass) at metamorphosis. Differences in survival between treatments were compared using a survival analysis Logrank Mantel-Cox test and displayed as a Kaplan-Meier survival curve. Time to metamorphosis and body size at metamorphosis were compared using one-way ANOVAs. Growth rates between treatments were compared using a repeated measure ANOVA. All analyses were conducted using Statview version 5.0.1. (SAS Institution Inc., North Carolina, USA).

## Results

### Effects of Salinity on Tadpole Survival

**Figure 1:**
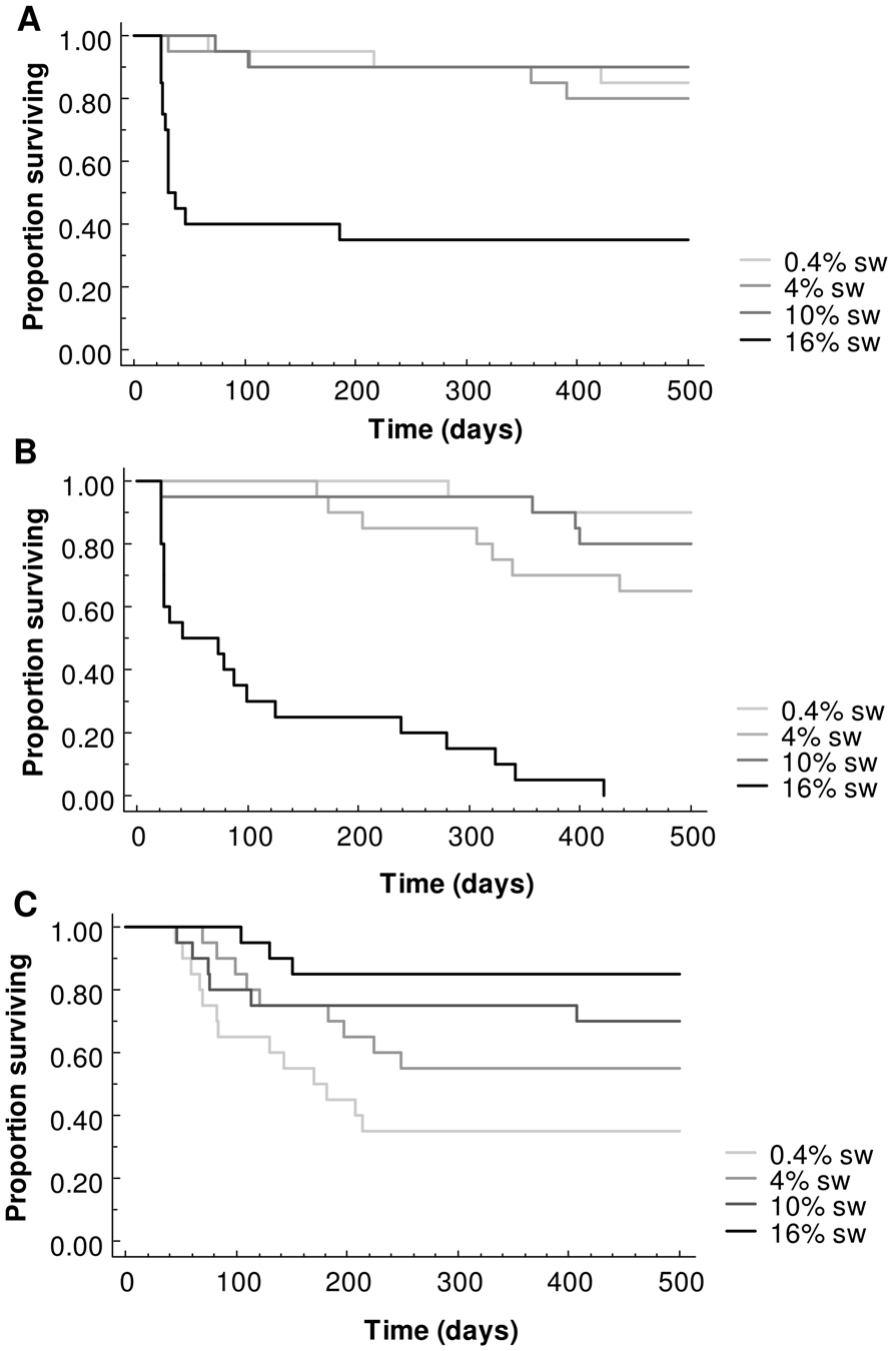
Proportion of tadpoles surviving from the effects of varying salinity over time for A *Limnodynastes tasmaniensis* B *Neobatrachus sudelli* C *Litoria aurea.*

Survival to metamorphosis of *L. tasmaniensis* tadpoles in the 16% sw treatment group was significantly lower (35% survival) than in all other treatment groups (Logrank Mantel-Cox: 0.4% sw χ^2^ = 14.159, p<0.01; 4% sw χ^2^ = 14.585, p<0.01; 10% sw χ^2^ = 13.067, p<0.01) (Table 1, Fig. 1a). Survival in all other groups was higher than 80%. In the 16% sw treatment group, most tadpole deaths (84.61%, 11/13) occurred within the first 30 days of the treatment.

**Table 1:** Mean time to metamorphosis, mean mass at metamorphosis, mean snout-vent length at metamorphosis and percentage survival of metamorphs under varying salinity treatments (mean±s.e.), n = 20 for all groups unless otherwise indicated.

Species	Treatment	Mean time to metamorphosis (days)	Survival (%)	Mean mass at metamorphosis (g)	Mean snout-vent length at metamorphosis (mm)
*Limnodynastes tasmaniensis*	0.4% sw#	105±4^ac^	85^a^	0.67±0.02	15.53±0.29
	4% sw	174±18^b^	80^a^	0.65±0.02	15.63±0.43
	10% sw	122±11^ac^	90^a^	0.79±0.04	16.74±0.35
	16% sw	143±29abc	35^b^	0.82±0.22	16.66±1.19
*Neobatrachus sudelli*	0.4% sw	292±22	90^a^	1.09±0.10	18.03±0.51
	4% sw	275±2	70^a^	1.07±0.07	17.49±0.42
	10% sw	338±22	80^a^	1.37±0.08	18.53±0.60
	16% sw	NA	0^b^	NA	NA
*Litoria aurea*	0.4% sw	164±10^a^	35^a^	1.56±0.13	22.54±0.56^a^
	4% sw	214±14^b^	55^b^	1.63±0.14	23.10±0.60^ab^
	10% sw	185±10^ab^	70^b^	1.80±0.08	24.25±0.45^bc^
	16% sw	186±7^ab^	85^b^	1.97±0.48	25.16±0.44^c^

abc Treatments with different superscript letters are significantly different to each other.

# n = 19.

In *N. sudelli*, total mortality was observed in the 16% treatment group, and as such, survival was significantly lower than all other groups (Logrank Mantel-Cox: 0.4% sw χ^2^ = 33.627, p<0.01; 4% sw χ^2^ = 21.252, p<0.01; 10% sw χ^2^ = 31.187, p<0.01) (Table 1, Fig. 1b).

Survival of *L. aurea* tadpoles was significantly lower in the 0.4% sw treatment group (35%) when compared to all other treatment groups (Logrank Mantel-Cox: 4% sw: χ^2^  =  5.795, p<0.05, 10% sw: χ^2^ = 5.322 p<0.051, 16% sw: χ^2^ = 10.196 p<0.05) (Table 1, Fig. 1c). The highest survival (85%) was observed in the 16% sw treatment group.

### Effect of Salinity on Development

For *L. tasmaniensis* and *L. aurea,* total time to metamorphosis was significantly different between treatment groups (ANOVA: *L. tasmaniensis* F_3,53_ = 1.956 p<0.01; *L. aurea* F_3,45_ = 3.051, p<0.05). One *L. tasmaniensis* tadpole in the 0.4% treatment group was excluded from analysis as it had neither metamorphosed nor died after 500 days of treatment.

*L. tasmaniensis* tadpoles in the 4% sw treatment took the longest to complete metamorphosis (mean = 174±18 days, n = 16). This was significantly longer than those in the 0.4% sw and the 10% sw groups, but not the 16% sw treatment group (Table 1). Growth rates over the first ten weeks of development were significantly different in all groups (Repeated measures ANOVA: F_3,53_ = 3.642, p = 0.018), with the fastest average growth rate observed in the 0.4% and 10% treatment groups (0.4% 3.956±0.082 mm/week; 4% 3.395±0.115 mm/week).

*L. aurea* tadpoles in the 4% sw treatment group took the longest to metamorphose (mean  =  214±14 days, n = 11), as was the case for *L. tasmaniensis*. This was significantly longer than the 0.4% sw treatment group, but not the 10% sw group, nor the 16% sw treatment group (Table 1). Salinity did not significantly effect the growth rate over the first 10 weeks of development in *L aurea* (Repeated measures ANOVA: F_3,45_ = 2.265, p = 0.094).

There were no differences in time to metamorphosis or growth rate over the first 10 weeks of development between treatment groups in *N. sudelli* (Table 1).

### Body Mass and Size at Final Metamorphosis

Salinity did not significantly affect the final body mass at metamorphosis of any species (ANOVA: *L. tasmaniensis;* F_3.53_ = 1.833 p = 0.15; *N. sudelli;* F_2.44_ = 2.827 p = 0.07; *L. aurea;* F_3,45_ = 2.371, p = 0.08), but snout vent length was significantly different in *L. aurea* (ANOVA: *Litoria aurea;* F_3,45_ = 5.009 p<0.01). *L. aurea* tadpoles in the 16% sw treatment group were significantly larger (mean  = 25.163±0.443 mm) than those in both the 0.4% sw and 4% sw treatment groups. In addition, tadpoles in the 10% sw treatment group were significantly larger (mean  = 24.251±0.447 1 mm) than those in the in the 0.4% sw treatment group (Table 1).

### Body Deformities

During development, several deformities were noticed in some individuals. Tail crimps or kinks [cf. 7] were noticed in individuals from the 0.4% sw (3/20), 10% sw (3/20) and 16% sw (1/20) treatments in *L. tasmaniensis,* and the 10% sw (1/20) and 16% sw (2/20) treatments in *L. aurea* However, all but one (1/10) of these individuals were still able to successfully complete metamorphosis.

## Discussion

Secondary salinisation has been suggested as an important factor contributing to anuran decline in Australia, but empirical data demonstrating negative fitness impacts remains limited. This study examined the effects of elevated salinity on growth, metamorphosis and survival of the tadpoles of three species of native Australian frog. Our results show the potential for elevated salinity to act as an agent of selection in native anuran species, with each species studied here showing a marked difference in survival under differing salinity treatments.

A previous study on *L. aurea* tadpoles from a coastal population (Kurnell NSW, Australia, 34°00′ S, 151°12′ E, 1998-9) reported that tadpoles tolerated salinities of up to 4% sw without apparent adverse effects, but showed a salinity threshold (above which significant mortality occurred) somewhere between 5.5% and 10% sw [8]. In contrast, our results showed a significantly higher survival in all salinities above 4% sw, and a lower survival in freshwater. There are several explanations for this contrast in findings including increased phenotypic plasticity, local adaptation or lineage adaptation in response to elevated levels of salinity in our experimental animals. Adaptation to salinity has previously been reported in other non-Australian amphibians. For example Gomez-Mestre *et. al* [23], [24] provided evidence for elevated salt tolerance of the embryonic and larval stages of populations of natterjack toads (*Bufo calamita*) inhabiting brackish ponds in southern Spain. However, in these cases adapted individuals transplanted to freshwater environments showed similar survival probabilities, length of larval period, and mass at metamorphosis compared to those native to freshwater. To our knowledge our study is the first report of anurans reared in freshwater having a lower survival than animals of the same population reared in brackish conditions. However, loss of performance of stress tolerant individuals in non-stressful environments has been demonstrated in other organisms such as bacteria [25], [26], and fish [27]. In order to identify the underlying mechanisms involved in the increased larval salinity tolerances shown here further studies comparing the effects of salinity on tadpoles of *L. aurea* from various populations along a salinity gradient will be required. Because differences in a mean phenotype among populations must have a genetic basis to be considered adaptive [28], conducting reciprocal transplant experiments would also be a valuable next step towards elucidating the adaptive response of *L. aurea* to challenging osmotic environments caused by secondary salinisation [29].

When compared to *L. aurea, L. tasmaniens*is and *N. sudelli* were less tolerant of high salinity, with both species showing a reduction in survival at salinities above 10% sw. Of the deaths that occurred in the 16% sw treatment groups, 85% of *L. tasmaniensis* and 45% of *N. sudelli* occurred within the first 30 days of treatment. In other species, newly hatched tadpoles have been demonstrated to be more vulnerable to increases in salinity compared to older cohorts *(e.g. Fejervarya cancrivora* [30], *Litoria ewingii* [7]). This vulnerability has been linked to differences in gill development [31] and kidney function [32] between cohorts.

Previous studies of a range of anurans (e.g. *R. cancrivora, B. calamita, L. ewingii,*) have reported that high salinity levels can retard tadpole development [7], [23], [31]. Our results indicate that when salinity rises above a tolerable concentration (in this case 10% sw), tadpoles of *L. tasmaniensis* accelerate metamorphosis instead of depending on weak osmoregulatory responses. *L. aurea* showed a similar pattern of accelerated development in the environment with the lowest prospective survival (in this case the 0.4% sw treatment group) when compared to the environment with intermediate survivals (4% sw). Our results suggest that these some species can invest energy in development when exposed to unfavourable salinities, be they too high or too low. Accelerating metamorphosis in unfavourable environments is expected to be costly, and in other anuran species studied rapid development typically results a reduction of body size at metamorphosis [7], [23]. While we observed a reduction in snout-vent length in *L. aurea* in freshwater, there were no other significant differences in weight or size in any other treatments or species. The tail crimps or kinks we observed in the 0.4% sw, 10% sw and 16% sw treatments in *L. tasmaniensis* and the 10% sw and 16% sw treatments in *L. aurea* may be indicators of an additional cost associated with increasing development. While not lethal in laboratory conditions, these tail crimps may affect a tadpole’s ability to effectively forage and evade predation [33].

Our study shows that elevated salinity negatively impacts on survival of *N. sudelli* and negatively on the survival, growth and development of *L. tasmaniensis* tadpoles, indicating that the salinisation of natural freshwater breeding habitats could contribute to the decline of local populations. In contrast, *L. aurea* tadpoles displayed a higher salinity tolerance, and this was higher than previously reported for this species, indicating this species may have the capacity to adapt to elevated environmental salinity. However, because survival of *L. aurea* was low in freshwater treatments, adaptation to elevated salinity may be associated with a loss of function under low saline conditions. Testing salinity tolerance of tadpoles from populations experiencing a range of salt conditions, running transplant experiments, and examining changes in osmoregulatory function will be needed to advance our understanding of the physiological and evolutionary responses of Australian anurans to secondary salinisation. This knowledge will help to determine how amphibians adapt to threatening environmental conditions, and will potentially assist with the management of the world’s declining frog populations.
